# Outcomes of Intraductal Placement of Covered Metal Stents for Unresectable Distal Malignant Biliary Obstruction

**DOI:** 10.3390/jcm12052001

**Published:** 2023-03-02

**Authors:** Manabu Yamada, Tsuyoshi Takeda, Takashi Sasaki, Takeshi Okamoto, Tsuyoshi Hamada, Takahiro Ishitsuka, Hiroki Nakagawa, Takafumi Mie, Takaaki Furukawa, Akiyoshi Kasuga, Masato Matsuyama, Masato Ozaka, Hideki Kobara, Tsutomu Masaki, Naoki Sasahira

**Affiliations:** 1 Department of Hepato-Biliary-Pancreatic Medicine, Cancer Institute Hospital of Japanese Foundation for Cancer Research, Tokyo 135-8550, Japan; 2 Department of Gastroenterology and Neurology, Faculty of Medicine, Kagawa University, Kita 761-0793, Japan; 3 Department of Gastroenterology, Graduate School of Medicine, The University of Tokyo, Tokyo 113-8655, Japan

**Keywords:** endoscopic biliary stenting, inside stent, malignant biliary obstruction, self-expandable metal stent

## Abstract

Intraductal self-expandable metal stent (SEMS) placement may prolong stent patency by reducing duodenobiliary reflux. This study aimed to evaluate the efficacy and safety of this biliary drainage method in patients with unresectable distal malignant biliary obstruction (MBO). Consecutive patients with unresectable MBO who underwent initial covered SEMS placement between 2015 and 2022 were retrospectively reviewed. We compared the causes of recurrent biliary obstruction (RBO), time to RBO (TRBO), adverse events (AEs), and reintervention rates between two biliary drainage methods (SEMSs placed above and across the papilla). A total of 86 patients were included (above: 38 and across: 48). Overall RBO rates (24% vs. 44%, *p* = 0.069) and median TRBO (11.6 months vs. 9.8 months, *p* = 0.189) were not significantly different between the two groups. The frequency of overall AEs was similar between the two groups in the entire cohort, but was significantly lower in patients with non-pancreatic cancer (6% vs. 44%, *p* = 0.035). Reintervention was successfully performed in the majority of patients in both groups. Intraductal SEMS placement was not associated with a prolonged TRBO in this study. Larger studies are warranted to further evaluate the benefit of intraductal SEMS placement.

## 1. Introduction

The endoscopic placement of self-expandable metal stents (SEMSs) is the standard palliative treatment for unresectable distal malignant biliary obstruction (MBO) due to longer stent patency compared to plastic stents [1,2]. Covered SEMSs (CMSs) may prolong stent patency compared to uncovered SEMS by preventing tumor ingrowth and can be removed at the time of reintervention. A recent meta-analysis reported the superiority of CMS over uncovered SEMSs for the treatment of distal MBO, especially in patients with pancreatic cancer [3]. However, recurrent biliary obstruction (RBO) due to stent migration, sludge formation, and food impaction remain unresolved issues associated with CMS. Duodenobiliary reflux through an SEMS placed across the papilla is considered a predisposing factor for sludge formation and cholangitis [4,5].

The intraductal placement of biliary stents (placed above the papilla) may be associated with longer stent patency and lower occlusion rate by reducing duodenobiliary reflux [6]. Few studies have evaluated the role of the intraductal placement of SEMS for unresectable distal MBO [4,7,8,9,10]. Two of these studies have compared the efficacy between biliary SEMS placed above and across the papilla, with conflicting results [9,10]. The intraductal placement of SEMS was not associated with a prolonged stent patency in one study [9], while it was associated with a longer stent patency in the other study [10]. However, different stent types were used in the two groups of the latter study; uncovered SEMSs were mainly placed above the papilla, while CMSs were mainly placed across the papilla.

The impact of endoscopic sphincterotomy (EST) with respect to the efficacy and safety of intraductal SEMS placement is an important matter of debate. Intraductal SEMS placement without EST may prolong stent patency by reducing duodenobiliary reflux, albeit with limited evidence [8,9,10]. A recent meta-analysis, which evaluated adverse events (AEs) after SEMS placement in patients with MBO, reported that the rates of early AEs (cholangitis and bleeding) were significantly lower in patients who did not undergo EST, while the rates of post-endoscopic retrograde pancreatitis were not significantly different between those who underwent EST and those who did not [11]. However, whether the results can also apply to intraductal SEMS placement is uncertain and avoiding EST may make reintervention more difficult and lead to more AEs.

In this study, we evaluated the efficacy and safety of CMS placement above the papilla in comparison with CMS placement across the papilla.

## 2. Materials and Methods

### 2.1. Patients

We conducted a retrospective study of consecutive patients with unresectable distal MBO who underwent initial CMS placement at our institution between June 2015 and May 2022. Only cases with at least 3 cm between the distal end of the stricture and the papilla were included in this study, as intraductal CMS placement was mainly performed in this situation. Excluded patients were as follows: (1) patients who had a history of more than one biliary SEMS placement; (2) patients who received an uncovered SEMS or a SEMS with an anti-reflux valve as the initial SEMS; (3) patients with surgically altered anatomy; and (4) patients with concomitant hilar biliary obstruction. The method of SEMS placement (above or across the papilla) was left to the endoscopist’s discretion. Written informed consent for the procedure was obtained from all patients. This study was approved by the ethics committee of our institution (Institutional Review Board number: 2022-GB-113). 

### 2.2. Endoscopic Interventions

Endoscopic retrograde cholangiopancreatography was performed using a therapeutic duodenoscope (JF260V, TJF260V, TJF-Q290V; Olympus Medical Systems, Tokyo, Japan) under conscious sedation. EST was generally performed in all patients who received an SEMS placed across the papilla, while it was less frequently performed in patients who received intraductal SEMS placement to reduce duodenobiliary reflux. An SEMS was deployed either above or across the papilla under fluoroscopic and endoscopic guidance. The length of the SEMS was selected based on cholangiographic findings.

The fully covered SEMSs used in this study were as follows: HANAROSTENT Biliary (M.I.Tech, Soul, Republic of Korea), Evolution Biliary Controlled-Release Stent–Fully Covered (Cook Medical, Bloomington, IN, USA), Niti-S SUPREMO stent (TaeWoong Medical, Soul, Republic of Korea), EGIS biliary stent (SB-Kawasumi Laboratories Inc., Kanagawa, Japan), and BONASTENT M-Intraductal (Standard Sci Tech, Soul, Republic of Korea).

### 2.3. Outcomes and Definitions

The primary outcome was time to RBO (TRBO). The secondary outcomes were technical success, clinical success, causes of RBO, AEs, reintervention, and overall survival (OS). Each outcome was generally defined according to the Tokyo Criteria 2014 [12]. RBO was defined as a composite endpoint of stent occlusion, stent migration, stent kinking, or non-occlusion cholangitis in cases where endoscopic biliary drainage was necessary to treat cholangitis. TRBO was defined as the time from the SEMS placement until RBO occurrence. Technical success was defined as the successful placement of an SEMS at the intended location, while clinical success was defined as a reduction (≥50%) or normalization in the serum bilirubin level within 2 weeks after SEMS placement, or no deterioration of the serum bilirubin level when the preprocedural value was normal. The severity of AEs was graded according to the American Society of Gastrointestinal Endoscopy lexicon guidelines [13]. Follow-up data were confirmed until 31 October 2022.

### 2.4. Statistical Analysis

Continuous variables are presented as median with ranges, and were compared using Mann–Whitney U test. Categorical variables are expressed as absolute numbers with proportions, and were compared using the χ^2^ test or Fisher’s exact test as appropriate. TRBO and OS were calculated using the Kaplan–Meier analysis and were compared using the log-rank test. The cumulative incidence of RBO was estimated using the competing risk analysis and were compared using Gray’s test [14]. Stent removal due to AEs and death without RBO were considered competing events. Statistical tests were two-sided and *p*-values < 0.05 were considered statistically significant. All statistical analyses were performed using the EZR software version 1.40 [15].

## 3. Results

### 3.1. Patient Characteristics

**Figure 1:**
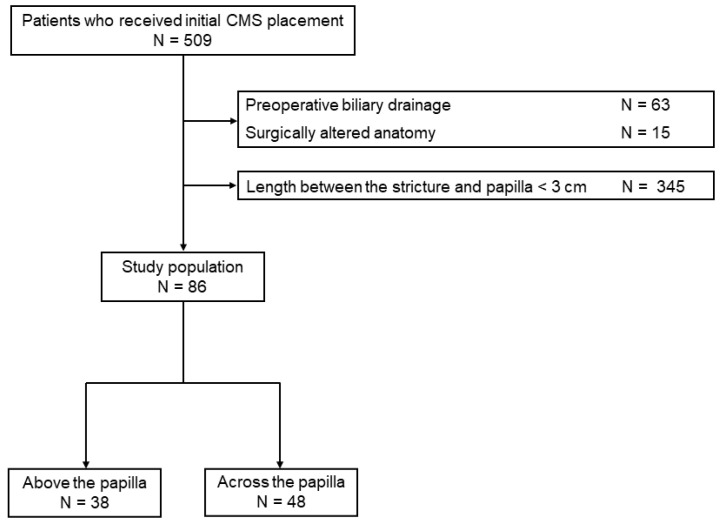
Patient flowchart.

A total of 509 patients underwent initial CMS placement for distal MBO during the study period. Of these, patients who underwent preoperative biliary drainage (*n* = 63), patients with surgically altered anatomy (*n* = 15), and cases in which the length between the distal end of the stricture and the papilla was less than 3 cm (*n* = 345) were excluded from the analysis. The remaining 86 patients were enrolled in this study (Figure 1). The most common cause of MBO was pancreatic cancer (*n* = 60), followed by biliary tract cancer (*n* = 14), colorectal cancer (*n* = 6), hepatocellular carcinoma (*n* = 1), hepatic cystadenocarcinoma (*n* = 1), gastric cancer (*n* = 1), small intestinal cancer (*n* = 1), uterine cancer (*n* = 1), and carcinoma of unknown primary (*n* = 1). Thirty-eight patients received intraductal CMS placement (the above group) and 48 patients received CMS across the papilla (the across group). 

Baseline and procedural characteristics of the two groups are summarized in Table 1. The proportions of pancreatic cancer (55% vs. 81%, *p* = 0.017) and tumor invasion of the main pancreatic duct (18% vs. 71%, *p* < 0.001) were significantly lower in the above group. Patients in the above group had more frequently received a duodenal stent (8% vs. 0%, *p* = 0.082) and undergone cholecystectomy (13% vs. 2%, *p* = 0.083) than those in the across group, although the difference was not statistically significant. Other baseline characteristics including age, sex, performance status, length of the stricture, length between the distal end of the stricture and the papilla, tumor status, presence of moderate to severe ascites, tumor invasion of the cystic duct orifice, and history of endoscopic biliary drainage before SEMS placement were not different between the two groups. Of the 34 patients who had undergone endoscopic biliary drainage before the SEMS placement in the above group, 25 underwent ENBD placement at our hospital (severe jaundice 15, cholangitis 7, and undiagnosed biliary stricture 3) and 9 underwent plastic stent placement at the referring hospital. On the other hand, of the 38 patients who had undergone endoscopic biliary drainage before SEMS placement in the across group, 36 underwent ENBD placement at our hospital (severe jaundice 19, cholangitis 7, and undiagnosed biliary stricture 10) and 2 underwent plastic stent placement at the referring hospital. Stents with lengths of 3–5 cm were mainly used in the above group, while lengths of 6–8 cm were used in the across group. Stent types were also different between the two groups, with BONASTENT M-Intraductal mostly used in the above group and HANAROSTENT Biliary mostly used in the across group. Endoscopic sphincterotomy was less frequently performed in the above group (66% vs. 98%, *p* < 0.001). Reasons for EST in the above group were as follows: difficult biliary cannulation: 11, history of EST at the referring hospital: 8, for insertion of biopsy forceps, etc., into the bile duct: 2, history of obstructive pancreatitis due to pancreatic cancer: 1, and at endoscopist’s discretion: 3.

### 3.2. Outcome Measures

Outcomes of SEMSs are shown in Table 2. Technical and clinical success rates were similar between the two groups. The frequency of AEs was lower in the above group (all AEs, 11% vs. 23%, *p* = 0.161; pancreatitis, 3% vs. 13%, *p* = 0.127), although the difference was not statistically significant. Of the six patients who developed pancreatitis in the across group, five patients underwent SEMS removal, while the SEMS removal was not attempted in the patient who developed pancreatitis in the above group. Of the three patients who developed cholecystitis in both groups, one patient each underwent SEMS removal. Overall RBO rates were not statistically different between the two groups (24% vs. 44%, *p* = 0.069), with stent occlusion being the most frequent cause of RBO in both groups. Stent migration occurred in one patient and five patients in the above and across groups, respectively. Other reasons for RBO include non-occlusion cholangitis (one patient in the across group) and kinking (one patient each in both groups).

**Figure 2:**
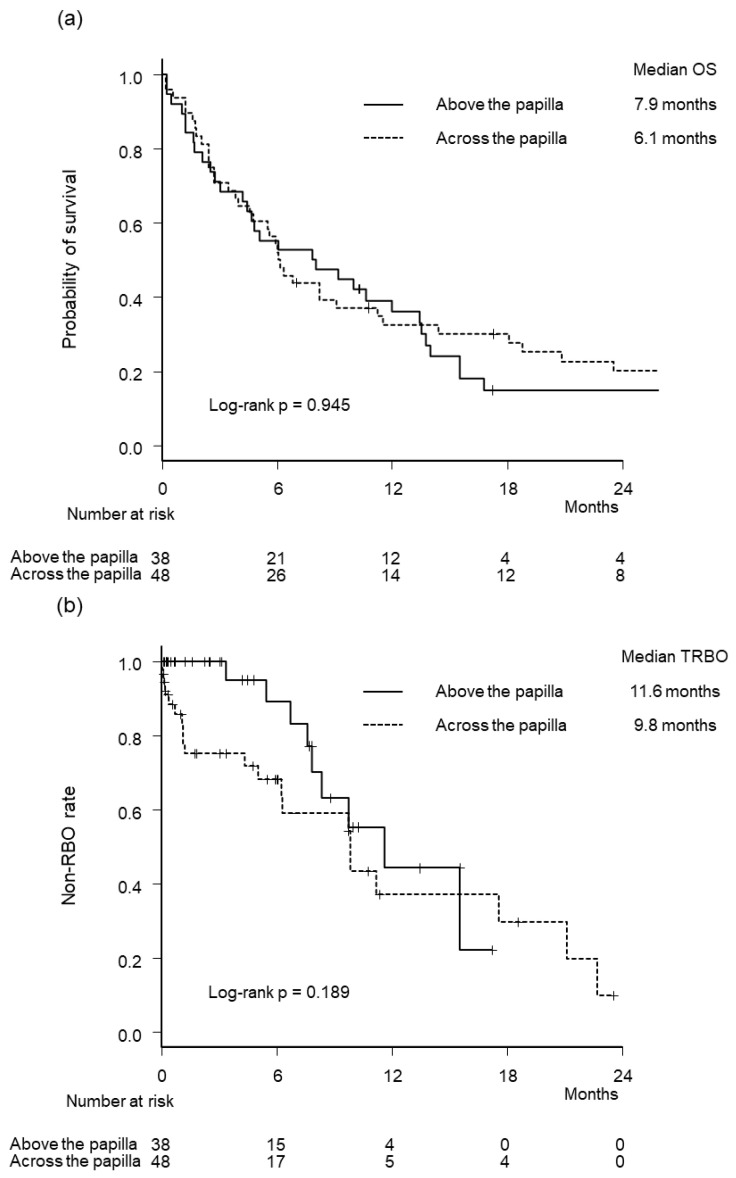
Kaplan–Meier curves by stenting methods. (**a**) Overall survival. (**b**) Time to recurrent biliary obstruction. RBO, recurrent biliary obstruction.

**Figure 3:**
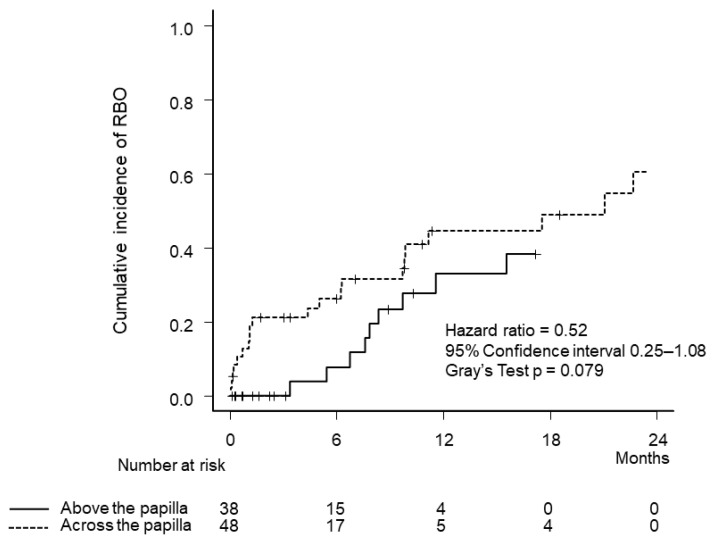
Cumulative incidence of recurrent biliary obstruction by stenting methods. RBO, recurrent biliary obstruction.

Kaplan–Meier curves of OS and TRBO are illustrated in Figure 2. Median OS (7.9 months vs. 6.1 months, *p* = 0.945) and TRBO (11.6 months vs. 9.8 months, *p* = 0.189) were not significantly different between the two groups. The cumulative incidence of RBO was lower in the above group (hazard ratio 0.52, 95% confidence interval, 0.25–1.08, *p* = 0.079), although the difference was not statistically significant (Figure 3).

### 3.3. Reinterventions

Endoscopic transpapillary reintervention was successful in all attempted cases (*n* = 9) in the above group. Four patients underwent the balloon sweeping of the bile duct without attempting stent removal. Stent removal was successful in four out of the five attempted cases; two cases underwent stent replacement above the papilla (CMS: 1 and plastic stent: 1), while the other two cases underwent stent replacement across the papilla (CMS: 1 and plastic stent: 1). In one patient whose stent could not be removed due to tumor overgrowth, a plastic stent was placed inside the SEMS, across the papilla.

Endoscopic transpapillary reintervention was successful in 20 out of 21 attempted cases (95%) in the across group. One patient underwent percutaneous transhepatic biliary drainage due to duodenal invasion at the superior duodenal angle. Five patients underwent the balloon sweeping of the bile duct without attempting stent removal. Stent removal was successful in 13 out of the 14 attempted cases; two cases underwent stent replacement above the papilla (CMS: 1 and uncovered SEMS: 1), eight cases underwent stent replacement across the papilla (CMS: 6 and plastic stent: 2), and three cases became stent-free due to temporary stricture resolution after chemotherapy (two of the three cases underwent concomitant radiation). Of the three cases that became stent-free, stricture recurred after two months in two cases and 12 months in one case. In two patients whose stents could not be removed due to tumor overgrowth, a plastic stent was placed inside the SEMS, across the papilla.

### 3.4. Subgroup Analysis

**Figure 4:**
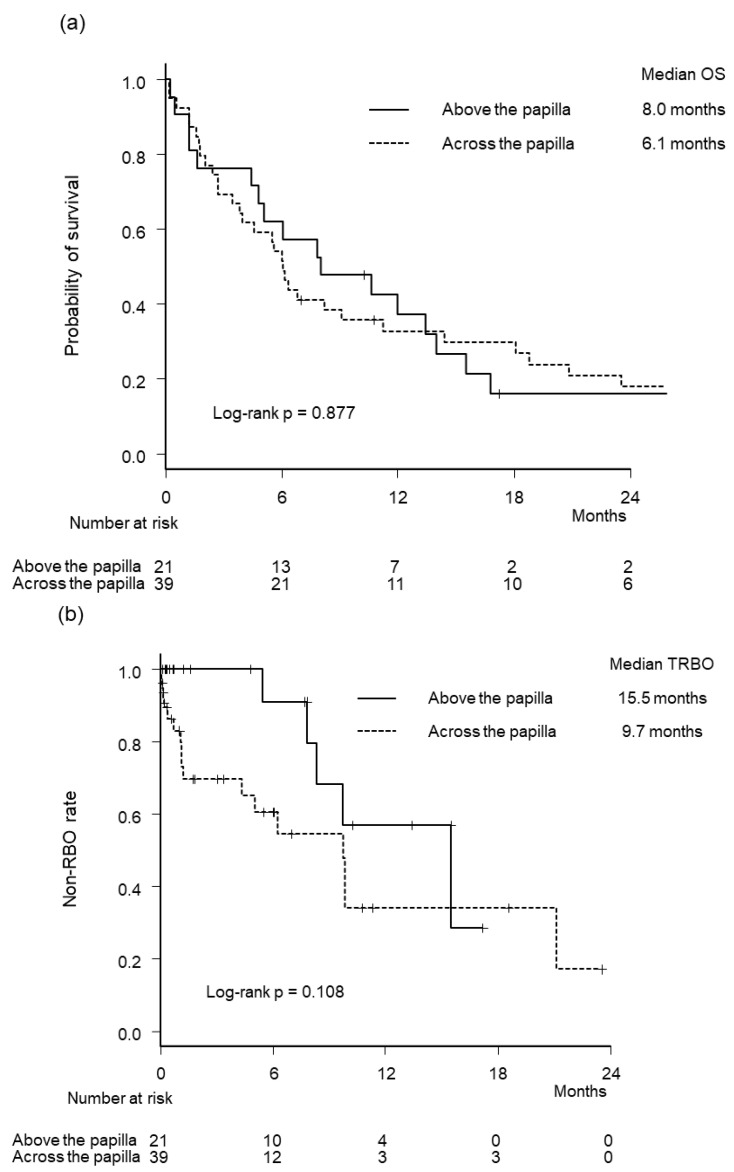

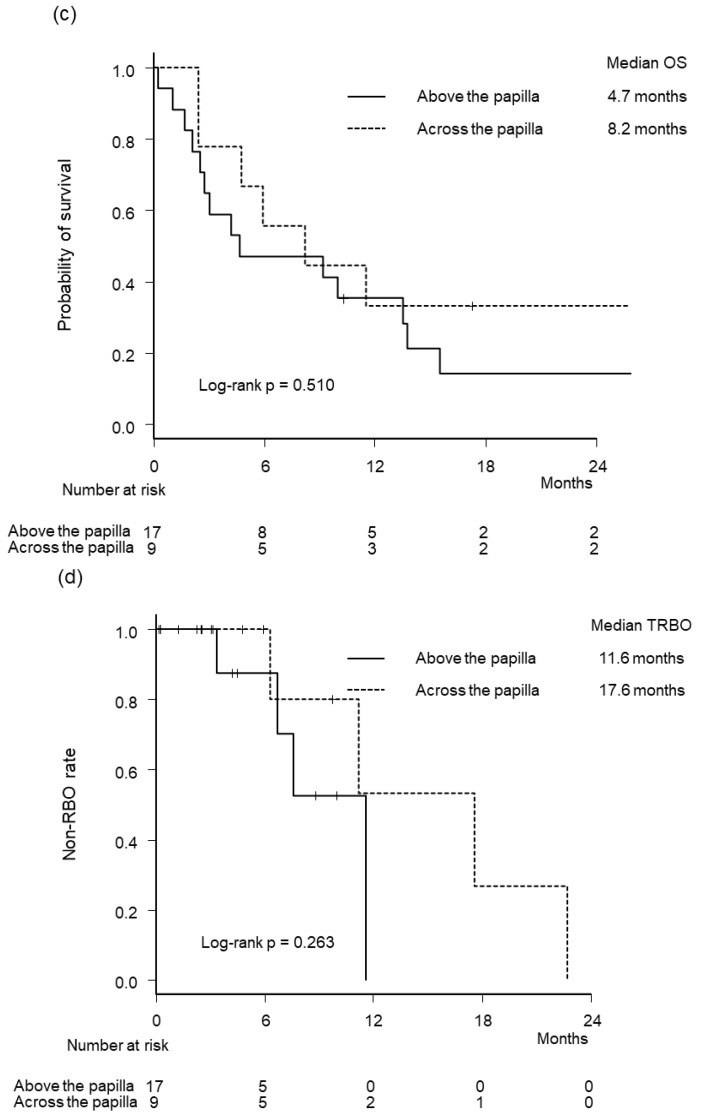
Kaplan–Meier curves by stenting method and primary disease type. (**a**) Overall survival in patients with pancreatic cancer. (**b**) Time to recurrent biliary obstruction in patients with pancreatic cancer. (**c**) Overall survival in patients with non-pancreatic cancer. (**d**) Time to recurrent biliary obstruction in patients with non-pancreatic cancer.

We further evaluated the efficacy and safety of intraductal CMS placement in patients with pancreatic cancer and non-pancreatic cancer because we speculated that the outcomes of intraductal CMS placement may differ between these two cancer types due to differences in bile duct axis deviation. Outcomes of SEMS stratified by primary disease type are shown in Table 3. Although the frequency of overall AE was similar between the two groups in patients with pancreatic cancer (14% vs. 18%, *p* > 0.999), it was significantly lower in the above group in patients with non-pancreatic cancer (6% vs. 44%, *p* = 0.035). Overall RBO rates were not statistically different between the two groups in patients with pancreatic cancer (24% vs. 44%, *p* = 0.166) and non-pancreatic cancer (24% vs. 44%, *p* = 0.382). Kaplan–Meier curves of OS and TRBO stratified by primary disease type are illustrated in Figure 4. Median OS and TRBO were not significantly different between the two groups in patients with pancreatic cancer and non-pancreatic cancer.

## 4. Discussion

The current study compared the efficacy and safety of two biliary drainage methods (the above and across groups) in patients with unresectable distal MBO. Although overall RBO rates (24% vs. 44%, *p* = 0.069) and the cumulative incidence of RBO (hazard ratio 0.52, 95% confidence interval, 0.25–1.08, *p* = 0.079) tended to be lower in the above group, intraductal SEMS placement was not associated with a prolonged TRBO (11.6 months vs. 9.8 months, *p* = 0.189). The frequency of overall AEs was similar between the two groups in the entire cohort, but was significantly lower in patients with non-pancreatic cancer (6% vs. 44%, *p* = 0.035). 

To date, SEMS placement across the papilla has been considered the standard biliary drainage method for unresectable distal MBO. As duodenobiliary reflux through the SEMS placed across the papilla remains a major cause of RBO, intraductal SEMS placement is a promising method that may prolong stent patency. However, studies comparing the two biliary drainage methods are scarce [9,10]. A randomized controlled trial of 84 patients comparing the efficacy of CMS placed above and across the papilla showed that intraductal SEMS placement was not associated with lower AE rates, lower stent occlusion rates (43.2% vs. 28.9%, *p* = 0.197), or longer stent patency (160 days vs. 191 days, *p* = 0.286) [9]. One possible reason for the negative results of the study is the short distance between the distal end of the stricture and the papilla (cases with lengths of at least 0.5 cm were included in that study), which may be too short for effective bile flow. Although we only included cases with at least 3 cm between the distal end of the stricture and the papilla, we also found that the overall RBO rates and median TRBO were not significantly different between the two biliary drainage methods. As intraductal SEMS placement without EST was reported to be associated with longer TRBO than intraductal SEMS placement with EST [10], the high frequency of EST (66% in the above group) in our study may have negatively affected TRBO in the above group. In this study, the majority of patients in the above group had undergone EST at the referring hospital or underwent EST at our hospital due to difficult biliary cannulation. On the other hand, a recent retrospective study of 73 patients comparing the efficacy of SEMS placed above and across the papilla showed that intraductal SEMS placement was associated with longer TRBO (307 days vs. 161 days, *p* = 0.022) [10]. However, that study included patients with different lengths between the distal end of the stricture and the papilla (2.3 cm vs. 0.5 cm, *p* < 0.001) and different types of SEMSs (uncovered SEMS/CMS, 25/5 vs. 7/36, *p* < 0.001). One of the strengths of our study is that patient characteristics including the length of the stricture and the distance between the distal end of the stricture and the papilla were similar between the two groups, with CMS used in all cases.

Another expected advantage of intraductal SEMS placement is the reduction in cholangitis (by reducing duodenobiliary reflux) and pancreatitis (by avoiding the obstruction of the main pancreatic duct orifice by the SEMS itself). Non-occlusion cholangitis (0% vs. 4%, *p* = 0.501) and pancreatitis (3% vs. 13%, *p* = 0.127) occurred less frequently in the above group, while the frequency of cholecystitis was similar between the two groups. Large prospective studies are needed to evaluate these expected benefits of intraductal SEMS placement.

Reintervention after the RBO of the initial SEMS is an important issue as the prognosis of patients with unresectable distal MBO has improved due to advances in chemotherapy. In this study, endoscopic reintervention was successful in most cases in both groups. Stent removal was successful in four out of the five patients in the above group by grasping the thread or the SEMS itself using a rat tooth forceps (EST was performed in three out of the four successful cases), but was unsuccessful in one case with tumor overgrowth. Use of an SEMS with a longer length may be an option to avoid tumor overgrowth.

Several issues relating to intraductal SEMS placement remain unresolved. First, the optimal indication for this biliary drainage method, including the desirable distance between the distal end of the stricture and the papilla, warrants further investigation. Second, the appropriate type of SEMS (fully covered, partially covered, or uncovered) is unclear. However, fully covered SEMS with a thread may be preferable to facilitate stent removal when necessary for reintervention. Finally, it is unclear whether EST should be performed. Theoretically, intraductal SEMS placement without EST may further prolong the stent patency by reducing duodenobiliary reflux, but this may make reintervention more difficult and lead to more AEs such as pancreatitis.

This study has several limitations. This was a single-center retrospective study with a limited number of cases. Although we included consecutive patients with at least 3 cm between the distal end of the stricture and the papilla in both groups, selection bias is inevitable. The decision to perform EST was left to the endoscopist’s discretion and some patients had undergone EST at the referring hospital. Several types of fully covered SEMS with different mechanical properties (such as radial force and axial force) were used in this study.

## 5. Conclusions

In conclusion, intraductal SEMS placement was not associated with a prolonged TRBO in this study. As the overall RBO rate and the cumulative incidence of RBO was numerically lower in the above group, larger studies on the efficacy of intraductal SEMS placement are warranted.

## Figures and Tables

**Table 1:** Baseline and procedural characteristics of patients who received covered metal stent above the papilla and across the papilla.

		Above the Papilla *n* = 38	Across the Papilla *n* = 48	*p* Value
Age, years		71 (46–96)	70 (47–88)	0.537
Sex				0.823
	Male	13 (34%)	18 (38%)	
	Female	25 (66%)	30 (63%)	
Eastern Cooperative Oncology Group Performance status				0.126
	0	27 (71%)	35 (73%)	
	1	10 (26%)	7 (15%)	
	≥2	1 (3%)	6 (13%)	
Stricture length, mm		21 (8–41)	22 (12–40)	0.770
Length between the distal end of the stricture and the papilla, mm		34 (30–77)	34 (30–45)	0.786
Primary disease				0.017
	Pancreatic cancer	21 (55%)	39 (81%)	
	Others	17 (45%)	9 (19%)	
Tumor status				0.813
	Locally advanced	10 (26%)	14 (29%)	
	Metastatic/recurrent	28 (74%)	34 (71%)	
Duodenal invasion		5 (13%)	5 (10%)	0.744
Co-existing duodenal stent		3 (8%)	0	0.082
Moderate to severe ascites		0	2 (4%)	0.501
Peritoneal dissemination		9 (24%)	9 (19%)	0.603
Tumor invasion of the main pancreatic duct		7 (18%)	34 (71%)	<0.001
Post-cholecystectomy		5 (13%)	1 (2%)	0.083
Tumor invasion of the cystic duct orifice *		7 (18%)	8 (17%)	>0.999
History of endoscopic biliary drainage before SEMS placement		34 (89%)	38 (79%)	0.248
Stent diameter, mm	6–8/10	2 (5%)/36 (95%)	0/48 (100%)	0.192
Stent length, cm	3–5/6–8	34 (89%)/4 (11%)	1 (2%)/47 (98%)	<0.001
Stent type				
HANAROSTENT Biliary		10 (26%)	35 (73%)	<0.001
Evolution biliary Controlled release Stent—fully covered		2 (5%)	7 (15%)	0.288
Niti-S SUPREMO stent		5 (13%)	4 (8%)	0.500
EGIS biliary stent		0	1 (2%)	>0.999
BONASTENT M-Intraductal		21 (55%)	1 (2%)	<0.001
Endoscopic sphincterotomy		25 (66%)	47 (98%)	<0.001
Chemotherapy after SEMS		32 (84%)	40 (83%)	>0.999

Continuous variables are expressed as the median (range) and categorical variables are expressed as absolute numbers (proportions). * Denominators adjusted to exclude six patients who underwent cholecystectomy. SEMS, self-expandable metal stent.

**Table 2:** Outcomes of covered metal stents placed above and across the papilla.

	Above the Papilla *n* = 38	Across the Papilla *n* = 48	*p* Value
Technical success	38 (100%)	48 (100%)	>0.999
Clinical success	36 (95%)	48 (100%)	0.501
Adverse events	4 (11%)	11 (23%)	0.161
Pancreatitis	1 (3%)	6 (13%)	0.127
Mild/moderate/severe	0/0/1	1/3/2	
Cholecystitis	3 (8%)	3 (6%)	>0.999
Mild/moderate/severe	2/1/0	2/1/0	
Non-occlusion cholangitis	0	2 (4%)	0.501
Mild/moderate/severe	0/0/0	1/0/1	
Recurrent biliary obstruction	9 (24%)	21 (44%)	0.069
Causes of recurrent biliary obstruction			
Occlusion	7 (18%)	14 (29%)	0.316
Sludge	5 (13%)	12 (25%)	0.275
Tumor ingrowth	0	0	>0.999
Tumor overgrowth	2 (5%)	2 (4%)	>0.999
Migration	1 (3%)	5 (10%)	0.222
Inward migration	1 (3%)	1 (2%)	>0.999
Outward migration	0	4 (8%)	0.126
Non-occlusion cholangitis	0	1 (2%)	>0.999
Kinking	1 (3%)	1 (2%)	>0.999

Categorical variables are expressed as absolute numbers (proportions).

**Table 3:** Outcomes of covered metal stents placed above and across the papilla, stratified by primary disease type.

	Pancreatic Cancer			Non-Pancreatic Cancer		
	Above the Papilla *n* = 21	Across the Papilla *n* = 39	*p* Value	Above the Papilla *n* = 17	Across the Papilla *n* = 9	*p* Value
Technical success	21 (100%)	39 (100%)	>0.999	17 (100%)	9 (100%)	>0.999
Clinical success	19 (90%)	39 (100%)	0.537	17 (100%)	9 (100%)	>0.999
Adverse events	3 (14%)	7 (18%)	>0.999	1 (6%)	4 (44%)	0.035
Pancreatitis	1 (5%)	4 (10%)	0.649	0	2 (22%)	0.111
Mild/moderate/severe	0/0/1	1/1/2		0/0/0	0/2/0	
Cholecystitis	2 (10%)	2 (5%)	0.606	1 (6%)	1 (11%)	>0.999
Mild/moderate/severe	1/1/0	1/1/0		1/0/0	1/0/0	
Non-occlusion cholangitis	0	1 (3%)	>0.999	0	1 (11%)	0.346
Mild/moderate/severe	0/0/0	0/0/1		0/0/0	1/0/0	
RBO	5 (24%)	17 (44%)	0.166	4 (24%)	4 (44%)	0.382
Causes of RBO						
Occlusion	3 (14%)	10 (26%)	0.512	4 (24%)	4 (44%)	0.382
Sludge	3 (14%)	8 (21%)	0.731	2 (12%)	4 (44%)	0.138
Tumor ingrowth	0	0	>0.999	0	0	>0.999
Tumor overgrowth	0	2 (5%)	0.537	2 (12%)	0	0.529
Migration	1 (5%)	5 (13%)	0.412	0	0	>0.999
Inward migration	1 (5%)	1 (3%)	>0.999	0	0	>0.999
Outward migration	0	4 (10%)	0.287	0	0	>0.999
Non-occlusion cholangitis	0	1 (3%)	>0.999	0	0	>0.999
Kinking	1 (5%)	1 (3%)	>0.999	0	0	>0.999

Categorical variables are expressed as absolute numbers (proportions).

## Data Availability

The data used in this study are available upon reasonable request from the corresponding author.
